# Confidence resets reveal hierarchical adaptive learning in humans

**DOI:** 10.1371/journal.pcbi.1006972

**Published:** 2019-04-09

**Authors:** Micha Heilbron, Florent Meyniel

**Affiliations:** Cognitive Neuroimaging Unit / NeuroSpin center / Institute for Life Sciences Frédéric Joliot / Fundamental Research Division / Commissariat à l'Energie Atomique et aux énergies alternatives; INSERM, Université Paris-Sud; Université Paris-Saclay; Gif-sur-Yvette, France; Dartmouth College, UNITED STATES

## Abstract

Hierarchical processing is pervasive in the brain, but its computational significance for learning under uncertainty is disputed. On the one hand, hierarchical models provide an optimal framework and are becoming increasingly popular to study cognition. On the other hand, non-hierarchical (flat) models remain influential and can learn efficiently, even in uncertain and changing environments. Here, we show that previously proposed hallmarks of hierarchical learning, which relied on reports of learned quantities or choices in simple experiments, are insufficient to categorically distinguish hierarchical from flat models. Instead, we present a novel test which leverages a more complex task, whose hierarchical structure allows generalization between different statistics tracked in parallel. We use reports of confidence to quantitatively and qualitatively arbitrate between the two accounts of learning. Our results support the hierarchical learning framework, and demonstrate how confidence can be a useful metric in learning theory.

## Author summary

Learning and predicting in every-day life is made difficult by the fact that our world is both uncertain (e.g. will it rain tonight?) and changing (e.g. climate change shakes up weather). When a change occurs, what has been learned must be revised: learning should therefore be flexible. One possibility that ensures flexibility is to constantly forget about the remote past and to rely on recent observations. This solution is computationally cheap but effective, and is at the core of many popular learning algorithms. Another possibility is to monitor the occurrence of changes themselves, and revise what has been learned accordingly. This solution requires a hierarchical representation, in which some factors like changes modify other aspects of learning. This solution is computational more complicated but it allows more sophisticated inferences. Here, we provide a direct way to test experimentally whether or not learners use a hierarchical learning strategy. Our results show that humans revise their beliefs and the confidence they hold in their beliefs in a way that is only compatible with hierarchical inference. Our results contribute to the characterization of the putative algorithms our brain may use to learn, and the neural network models that may implement these algorithms.

## Introduction

In real-world environments, learning is made difficult by at least two types of uncertainty [1]. First, there is inherent uncertainty in many real-world processes. For instance, the arrival of your daily commute may not be perfectly predictable but subject to occasional delays. Faced with such random fluctuations, learners should integrate as many observations as possible in order to obtain a stable, accurate estimate of the statistics of interest (e.g. the probability of delay) [2,3]. Second, there is the higher-order uncertainty related to sudden changes in those very statistics (*change points*). For instance, engineering works may increase the probability of delay for an extended period. When faced with a change point, learners should discount older observations and rely on recent ones instead, in order to flexibly update their estimate [2,4,5].

Confronted with both forms of uncertainty, the optimal learning strategy is to track not only the statistic of interest but also the higher-order probability of change points. This enables learners to render their estimate stable when the environment is stable (i.e. between change points) and flexible when the environment changes [2,4,6–9]. Importantly, this approach assumes that learners use a *hierarchical generative model* of their environment. Such a model comprises multiple levels, of which lower levels depend on higher ones: current observations (level 1) are generated according to statistics of observations (level 2) which themselves may undergo change points (level 3). The hierarchical approach is widely used to study learning in both health [2,10] and disease [11,12]. However, efficient learning in dynamic environments is also possible *without* tracking higher-order factors such as the likelihood of individual change points [3,13–16], and a large body of work indeed uses such a solution to model behavioral and brain responses [17–19]. Computationally, this approach is very different as it assumes that learners do not take higher-level factors (e.g. change points) into account, and hence use a non-hierarchical or *flat* model of the world.

The possibility that the brain uses internal hierarchical models of the world is an active area of research in cognitive science [20], and has important consequences for neurobiology, since hierarchical models [21,22] and non-hierarchical ones [3,18] require different neural architectures. In learning theory however, internal hierarchical models pose somewhat of a conundrum, being simultaneously assumed critical by some frameworks for learning under uncertainty [2,4,8] and unnecessary by others [3,16–18,23].

One possible explanation for this conundrum is that the brain might resort to different learning algorithms in different situations. Here, we explore another explanation (compatible with the former): in many situations, flat approximations to hierarchical solutions are so efficient that both accounts become difficult to distinguish in practice. Indeed, previous studies using quantitative model comparison reported conflicting results: some authors found that learning was best explained by hierarchical models [10,11,24,25] while others found that flat models best explained their results [17,18,26].

Here, our goal is to provide an experimental learning situation in which learners demonstrably use a hierarchical model. We provide a task and a simple analysis that reliably tests whether learners use a hierarchical model of the world. Our test relies not just on comparing model fits, but also on detecting a *qualitative signature* or *hallmark* that is uniquely characteristic of an internal hierarchical model.

## Results

### Modulations of apparent learning rate are not a hallmark of hierarchical processing

**Fig 1:**
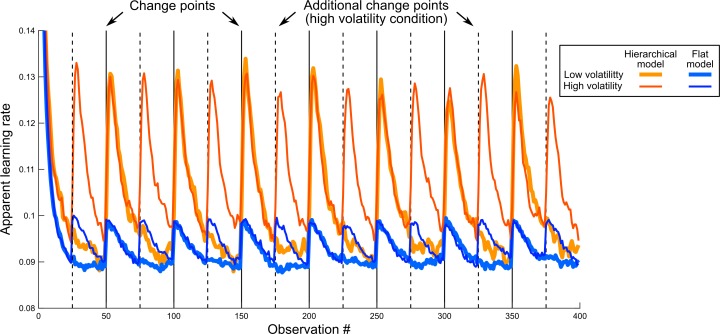
Apparent learning rate modulations in previous designs are not a hallmark of hierarchical processing. This simulation is inspired by a previous study by Behrens et al [2], in which the reward probability was not fixed but changed abruptly; the authors used different volatility levels (i.e. different numbers of change points). Similarly, we generated sequences with low volatility (7 change points, see vertical plain black lines), and high volatility (see additional change points, vertical dashed dashed lines). The sequences were binary (absence or presence of reward) and the reward probability was resampled randomly after each change point. We consider two learning models: a hierarchical model, which estimates the reward rate, taking into account the possibility of change points; and a flat model that computes the reward rate near-optimally based on a fixed leaky count of observations, and a prior count of 1 for either outcome (see Methods). Each model has a single free parameter (respectively, a priori volatility and leak factor) which we fit to return the best estimate of the actual generative reward probabilities in both the low and high volatility conditions together. Keeping those best fitting parameters equal across both conditions, we measured the dynamic of the apparent learning rates of the models, defined as the ratio between the current update of the reward estimate (θ_t+1_-θ_t_) and the prediction error leading to this update (y_t+1_-θ_t_). The hierarchical model shows a transient increase in its apparent learning rate whenever a change point occurs, reflecting that it gives more weight to the observations that follow a change point. Such a dynamic adjustment of the apparent learning rate was reported in humans [5]. The flat model showed a qualitatively similar effect, despite the leakiness of its count being fixed. Note that since there are more change points in the higher volatility condition (dashed lines), the average learning rates of both models also increase overall with volatility, as previously reported in humans [2]. The lines show mean values across 1000 simulations; s.e.m. was about the line thickness and therefore omitted.

By tracking the higher-order probability of change-points, learners using a hierarchical model can adjust their weighting of prior knowledge and new observations to the changeability of the environment. A highly influential result suggesting that human learners might apply this strategy demonstrated that the *apparent learning rate—*technically the ratio between the update size and the prediction error at any given observation—was modulated by change points in humans [2,5]. This was argued a hallmark of hierarchical processing, since increasing the learning rate after change points would only be expected from a hierarchical learner (tracking both the statistics of observations and changes in those statistics) and not from a flat learner (tracking only the statistics). However, we show a counter-example: a flat learning model whose parameters are kept fixed and nevertheless shows systematic modulations of the apparent learning rate without actually tracking the higher-level likelihood of change points (**Fig 1**, Methods and **Supplementary Results 2 in S1 File**). Although the modulations are smaller in the flat model than in the hierarchical one, they are qualitatively identical, demonstrating that such modulations are not uniquely characteristic of hierarchical models.

This counter-example suggests that the mere presence of apparent learning rate modulations is not sufficiently specific, and that a new qualitative test must tap into a different property in order to reveal a truly unique hallmark of hierarchical learning.

### Simulations suggest confidence can provide valuable information to discriminate models

When developing a new test, we first asked what quantity or metric the test should target. In earlier studies on learning under uncertainty, subjects tracked changing statistics such as the probability of reward or of a stimulus [2,10,17,25,27,28], or the mean of a physical quantity like the location or magnitude of reward [5,7]. Learning was then probed either from choices guided by the learned statistics [2,10,17,25,26] or using explicit reports of those statistics [5,7,8,27]. Both choices and explicit reports are *first-order metrics*, as they only reflect the estimated statistics themselves. However, since a first-order metric only describes the level of observations, and since all models aim at providing a good description of observations, a first-order metric may be seldom unique to a single model. By contrast, *second-order metrics*, such as the learner’s confidence about her estimates, also describe the learner’s inference and may convey additional information to discriminate models, not contained in the first-order estimate [29].

**Fig 2:**
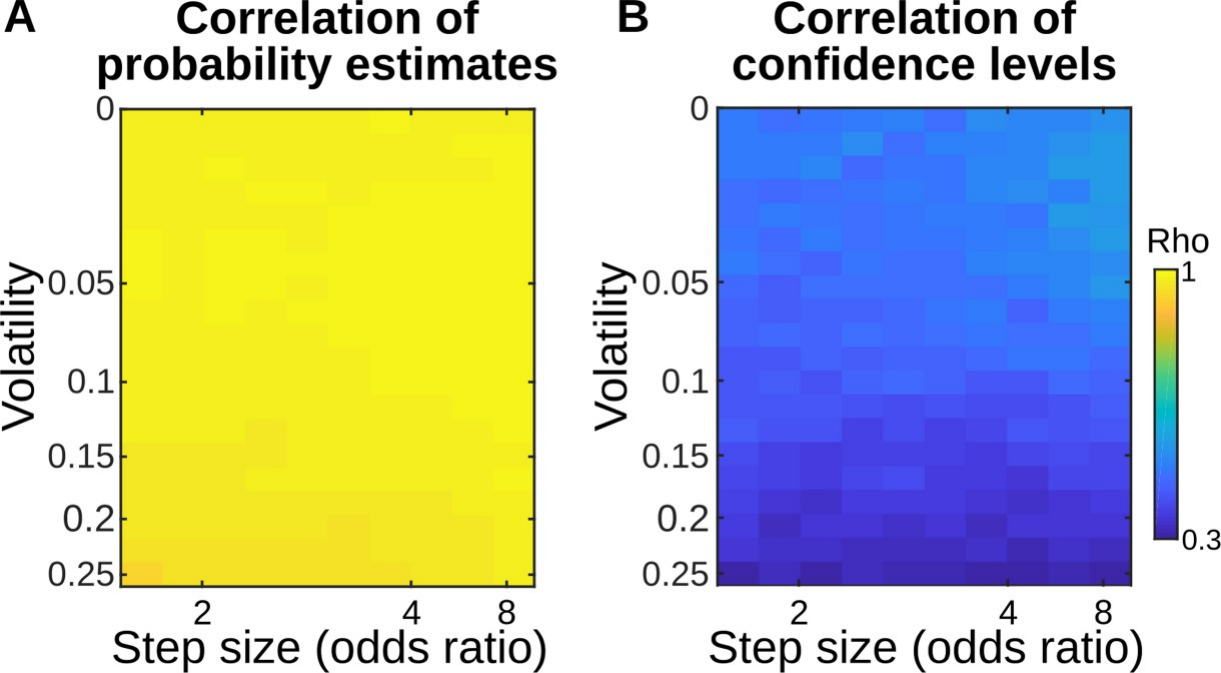
Correlation between the hierarchical and flat models in a classic probability learning task is higher for probability estimates than for confidence levels. We simulated a classic probability learning task, similar to the one by Behrens et al 2007. In this task, the binary observation made on each trial (e.g. presence or absence of reward) is governed by a probability that changes discontinuously at so-called change points. For the sake of generality, we varied the volatility (probability of a change point) and the step size of those changes (minimum fold change, in odds ratio, affecting the generative probability). For each combination of volatility and step size, we simulated 100 sequences to achieve stable results and we fit the single free parameter of each model (respectively, a priori volatility and leak factor) onto the actual generative probabilities of the observed stimuli in the sequences. The resulting parameterized models therefore return their best possible estimate of the hidden regularities, in each volatility-step size condition. We then simulated new sequences (again, 100 per condition) to measure **(A)** the correlation between the estimated probabilities of stimuli between the two models, and **(B)** the correlation (Pearson’s rho) between the confidence (log-precision) that those models entertained in those estimates. The correlations indicate that probability estimates are nearly indistinguishable between the two models, whereas their confidence levels are more different.

For illustration, we simulated a hierarchical model and a flat model (same models as in **Fig 1**). The latter is minimally different from the classic delta-rule, but extended so as to provide confidence levels (see Methods and **Supplementary Results 1 in S1 File**). We simulated a typical learning problem, where participants estimate a reward probability that unpredictably changes discontinuously over time. Over a large range of possible task parameters, the probability estimates of the optimal hierarchical model and a near-optimal flat model were highly correlated (Pearson ρ>0.9) whereas their confidence levels were much less correlated (see **Fig 2**and Methods). The same conclusion holds when simulating a more complex learning task that we present below (See panels **A-B in S1 Fig**). Note that while we used confidence, results should be similar when using other, related metrics (see Discussion).

Importantly, these results only reflect *average correlations* and do not speak to unique hallmarks or signatures. Rather, they simply show that even when first-order estimates of two models are nearly indistinguishable, their second-order metrics can be much less correlated, thus offering an additional source of information not often considered in learning theory.

### A task allowing for a more direct test for an internal hierarchical model

A hierarchical model is defined by its levels, the variables in each level and the dependencies between those variables. However, these aspects are often confounded in previous experiments because their task structure was quite simple. In a typical experimental situation [2,5,7,10,17,25,27,28] the observations received by the subject (level 1) are controlled by *only one statistic* (level 2), such as the probability of reward, whose value abruptly changes at change points (level 3). This task structure is hierarchical, but so simple that it takes the form of a linear chain of dependencies: at each level, a single variable depends on a single variable of the next level (see **panel A in S2 Fig**). With such a structure, the notions of hierarchy and changeability are confounded. This enables a learner that can cope with changeability, for instance by using leaky (and perhaps adjustable) integration [3,18], to learn effectively without needing an internal, hierarchical model of the task structure.

By contrast, using a more complex task structure gives rise to forms of inference and learning that are possible only if learners rely on an internal hierarchical model. Here, we build on a previously used task [8,24] in which two changing statistics are governed by the same change points. This breaks the pure linear structure of previous tasks by introducing a branch which renders its hierarchical nature more prominent, see **panel B in S2 Fig**. Crucially, in this situation, *only* a hierarchical model can leverage the coupling of multiple statistics arising from a common higher-order factor and generalize appropriately between the estimated statistics. Probing this generalization will be our test for hierarchy, as we will detail below.

**Fig 3:**
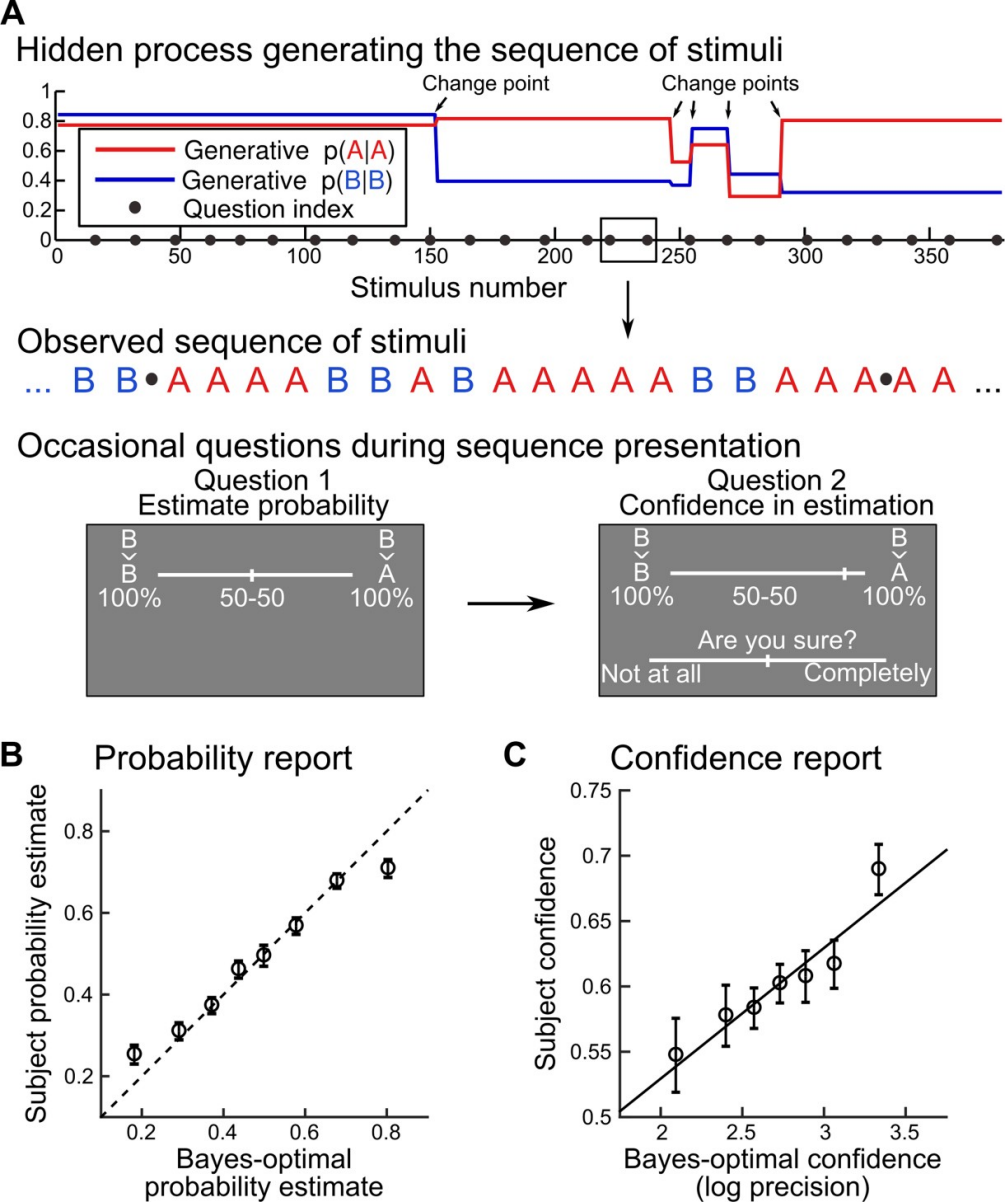
Behavioral task: Learning of dynamic transition probabilities with confidence reports. **(A)** Probability learning task. Human subjects (N = 23) were presented with random sequences of two stimuli, A and B. The stimuli were, in distinct blocks, either auditory or visual and they were perceived without ambiguity. At each trial, one of either stimulus was sampled according to a probability that depended on the identity of the previous stimulus: p(A_t_|A_t-1_) and P(B_t_|B_t-1_). These transition probabilities underwent occasional, abrupt changes (change points). A change point could occur at any trial with a probability that was fixed throughout the experiment. Subjects were instructed about this generative process and had to continuously estimate the (changing) transition probabilities given the observations received. Occasionally (see black dots in A), we probed their inferences by asking them, first, to report the probability of the next stimulus (i.e. report their estimate of the relevant transition probability) and second, to rate their confidence in this probability estimate. **(B, C)** Subjects’ responses were compared to the optimal Bayesian inference for this task. Numeric values of confidence differ between subjects and models since they are on different scales (from 0 to 1 in the former, in log-precision unit in the latter). For illustration, the optimal values were binned, the dashed line (B) denotes the identity, the plain line (C) is a linear fit, and data points correspond to subjects’ mean ± s.e.m.

In the task (see **Fig 3A**), participants observed long sequences of two stimuli (A and B), the occurrence of which was governed by two transition probabilities which subjects had to learn: p(A_t_|A_t-1_) and p(B_t_|B_t-1_). The value of each probability was independent, but at unpredictable moments (*change points*) both simultaneously changed. Subjects were informed about this generative process in advance. They passively observed the stimuli and were asked to report both their estimate of the transition probability leading to the next stimulus, and their confidence in this estimate.

### Probability and confidence estimates closely follow the hierarchical model

Before testing for an internal hierarchical model, we first wanted to verify whether subjects had performed the task well, in the sense that their responses were consistent with the hierarchical, Bayes-optimal solution. As a benchmark, we used the optimal model for this task; this model was not fitted onto subjects’ data, but set so as to optimally solve the task by ‘inverting’ its hierarchical generative structure using Bayes’ rule (see methods). As displayed in **Fig 3B and 3C**, linear regressions show an agreement between participants’ probability estimates and optimal probability estimates (defined as the mean of the posterior; regressions are computed at the subject-level: β = 0.66±0.06 s.e.m., t_22_ = 11.13, p = 1.7 10^−10^), and subjective confidence reports and optimal confidence (defined as the log precision of the posterior; β = 0.10±0.03 s.e.m., t_22_ = 3.06, p = 0.0058); for further checks of robustness, see **Supplementary Results 3 in S1 File**. In order to quantify the variance explained, we also computed the Pearson correlations corresponding to those regressions: ρ = 0.56±0.04 s.e.m. for probability estimates and ρ = 0.19±0.05 s.e.m. for confidence. Despite being somewhat noisier, confidence reports also showed many properties of optimal inference (see **Supplementary Results 4 in S1 File**).

Since we propose that subjects’ confidence reports can convey useful information over and above their first-order estimates, the next thing we verified was that confidence reports indeed conveyed information that was not already conveyed implicitly by the first-order estimates. We tested this in our data by regressing out the (theoretically expected) covariance between subjects’ confidence reports and several metrics derived from first-order estimates (see **Supplementary Results 4 in S1 File**); the residuals of this regression still co-varied with optimal confidence (β = 0.028±0.012, t_22_ = 2.3, p = 0.029). This result was replicated by repeating the analysis on another dataset [8]: β = 0.023±0.010, t_17_ = 2.2, p = 0.0436; and also in the control experiment detailed below: β = 0.015±0.006, t_20_ = 2.3, p = 0.034. These results indicate that subjective confidence and probability reports are not redundant, and thus that confidence is worth investigating.

Having verified that confidence and probability reports closely followed estimates of an optimal hierarchical model, and that both metrics were not redundant, we then tested whether subjects’ reports, overall, could not be better explained by a different, computationally less sophisticated model: the flat model introduced above (**Fig 1**, also see Methods and **Supplementary Results 1 in S1 File**), that approximates the full Bayesian model extremely well. Both models have the same number of free parameters, so model comparison (at least using standard comparison methods like BIC or AIC) boils down to comparing the goodness-of-fit. We first took the parameters that provide the best estimate of the true generative probabilities. The goodness-of-fit, assessed as mean square error (MSE) between subjects’ and models’ estimates, was better for the hierarchical model than for the flat model (paired difference of MSE, hierarchical minus flat: -0.0051±0.0014 s.e.m., t_22_ = -3.7, p = 0.0013). Note that subjects’ estimates of volatility, a key task parameter here, usually deviate from the optimum and show a large variability [5,30], which could bias our conclusion. We therefore fitted the model parameters per subject, and we found that the difference in fit was even more significant (-0.0077±0.0019 s.e.m., t_22_ = -3.97, p = 6.5 10^−4^). This result replicates a previous finding [24]. We then repeated the comparison for confidence levels. When model parameters were set to best estimate the true transition probabilities, the hierarchical model showed a trend toward a significantly lower MSE compared to the flat model (paired difference of MSE, hierarchical minus flat: -0.0017±0.0010 s.e.m., t_22_ = -1.8, p = 0.084). When model parameters were fitted onto each subjects’ confidence reports, this difference was significant (-0.0027±0.0012 s.e.m., t_22_ = -2.36, p = 0.028).

In sum, these results show that participants successfully performed the task and that the hierarchical model was quantitatively superior to the flat model in explaining subjects’ probability estimates and confidence ratings. This leaves us with the last and perhaps most important question: did subjects also show a *qualitative signature* that could only be explained by a hierarchical model?

### Subjective confidence reveals a hallmark of an internal hierarchical model

**Fig 4:**
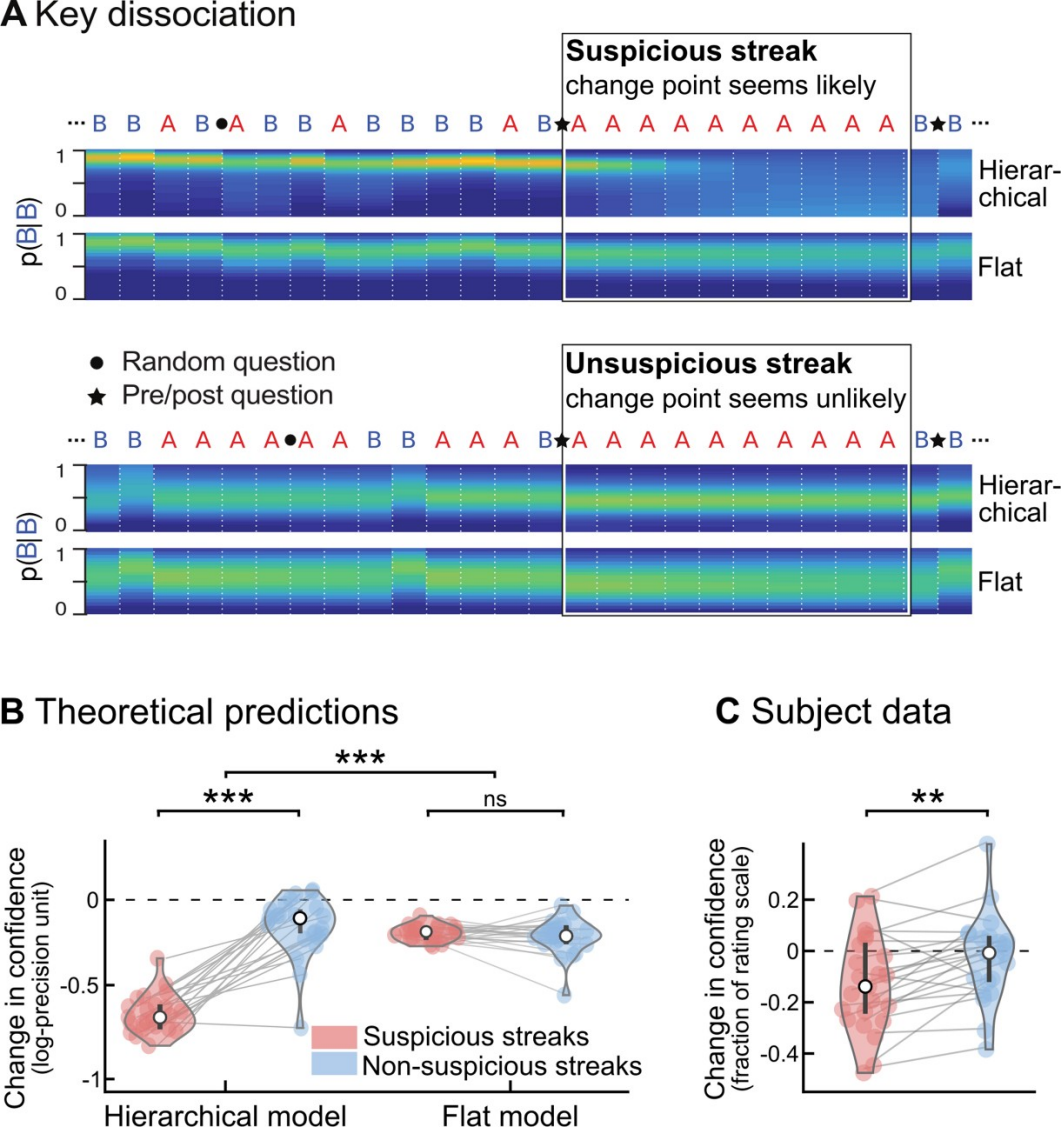
A qualitative signature of hierarchical learning in confidence reports. **(A)** Divergent predictions of hierarchical versus flat learning models. Two fragments of sequences are shown in which one stimulus (‘A’) is consecutively repeated 10 times. In the upper fragment, this streak of repetitions is highly unlikely (or ‘suspicious’) given the context, and may indicate that the underlying statistics changed. By contrast, in the lower fragment, the same streak is not unlikely, and does not suggest a change point. The heat maps show the posterior probability distribution of P(B|B), i.e. the probability of a repetition of the other stimulus (B), estimated by the hierarchical and flat models. In a hierarchical model, unlikely streaks arouse the suspicion of a global change in statistics, causing the model to become uncertain about its estimates of both transition probabilities, despite having acquired no direct evidence on P(B|B). In a flat model, by contrast, a suspicious streak of As will not similarly decrease the confidence in P(B|B), because a flat model does not track global change points. To test for this effect, pre/post questions (indicated by a star) were placed immediately before and after selected streaks, to obtain subjective estimates of the transition probability corresponding to the stimulus not observed during the streak. Streaks were categorized as suspicious if they aroused the suspicion of a change point from the hierarchical, Bayes-optimal viewpoint. Note that the flat model also shows a decrease in confidence, because it progressively forgets its estimates about P(B|B) during a streak of As, but, there is no difference between suspicious and non-suspicious streaks. **(B)** For the sequences presented to subjects, the change in confidence (post-streak minus pre-streak) was significantly modulated by streak type in the hierarchical model, but not in a flat model. **(C)** Subjects’ confidence showed an effect of streak type predicted by the optimal hierarchical model. As in Fig 4C, confidence values in subjects and models are on different scales. Error bars correspond to the inter-subject quartiles, distributions show subjects' data; significance levels correspond to paired t-test with p<0.005 (**) and p<^10–12^ (***).

Identifying the qualitative signature proposed here was possible because our task involves two transition probabilities, P(A|A) and P(B|B), whose changes were *coupled*, occurring simultaneously. In this context, a flat learner only estimates the value of each transition probability, while a hierarchical model *also* estimates the probability of a *global* change point. Faced with a global change point, the hierarchical learner then reacts optimally and makes its prior knowledge more malleable by becoming uncertain about *both* P(A|A) and P(B|B). Importantly, using this mechanism, an internal hierarchical model should allow for generalization: if a change point is suspected after observing just one type of transition (e.g. AAAAAAA, when P(A|A) was estimated to be low) a hierarchical learner would *also* become uncertain about the other quantity, P(B|B), despite having acquired no direct evidence on this transition (**Fig 4A**). Critically, this form of indirect inference is unique to hierarchical models and thus offers a powerful test of hierarchical theories of learning.

To test for this generalization effect, we focused on streaks of repetitions, and distinguished between streaks that seem unlikely in context and may signal a change point (*suspicious streaks)* and streaks that do not (*non-suspicious streaks*). Stimulus sequences were carefully selected to contain enough suspicious and non-suspicious streaks and to control for confounds such as streak duration (see Methods). Questions were inserted just before and after the streak, so that subjects reported their estimate of (and confidence in) the other, non-repeating transition (**Fig 4A**). Exact theoretical predictions for both models are found in **Fig 4B**. In the hierarchical model, confidence decreases strongly after suspicious, but much less after non-suspicious streaks. In the flat model, however, there is no such difference. Strikingly, subjective reports followed the hierarchical account (see **Fig 4C)**: confidence decreased strongly after suspicious (-0.12±0.04 s.e.m, t_22_ = -3.2, p = 0.004) but not after non-suspicious streaks (-0.02±0.03 s.e.m., t_22_ = -0.7, p = 0.51), and this interaction was significant (paired difference, 0.10±0.03 s.e.m., t_22_ = 3.7, p = 0.001).

### Various controls demonstrate the specificity of the effect on confidence

We now rule out a series of potential confounding explanations. First, one concern is that the analysis above uses models optimized to estimate the true transition probabilities of the task. However, our conclusions remain unaffected if we use models fitted onto each subject (**panels C, E in S3 Fig**). Another concern is that our analyses assume subjects were tracking transition probabilities, while they may in fact have been tracking another (heuristic) quantity, perhaps using a flat model. Detailed analysis revealed that subjects did in fact track transition probabilities (see **Supplementary Results 4 in S1 File**) and that no heuristic flat model could explain the selective decrease of confidence (**panels B, D, F in S3 Fig**). Finally, we also considered models that were technically hierarchical but that erroneously assume that the two transition probabilities have independent (rather than identical) change points (see panel C in **S2 Fig**). These models did not show the critical effect of streak type (**panels A, C, E in S3 Fig**), indicating that our test is diagnostic of the ability to transfer knowledge between dependent variables. This transfer is not afforded by all hierarchical models, but only those which entertain the correct, relevant dependencies.

One may also wonder whether the effect reported in **Fig 4** for confidence is also found in another variable. **S4 Fig** shows that probability estimates (the ones about which confidence is reported and shown in **Fig 4**) are not affected by streak types neither in subjects (paired difference between streak types, -0.01±- 0.02 s.e.m., t_22_ = -0.5, p = 0.59) nor in the hierarchical model (-0.01±0.01 s.e.m., t_22_ = -1.4, p = 0.17). A more subtle effect is that, when a change point is suspected, generalization should reset the estimate of the unobserved transition probability, which should thus get closer to the prior value 0.5. However, this effect is less straightforward, because the estimated transition probability may already be close to 0.5 before the streak. Indeed, even in the hierarchical, Bayes-optimal model, streak type had only a weak effect (paired difference, 0.02±0.01 sem, t_22_ = 2.9, p = 0.008), compared to the effect on confidence (**Fig 4,** t_22_ = 11.7, p = 6.9 10^−11^). The expectedly weaker effect of streak type on the distance to the prior was not detected in participants (-0.0036 +/- 0.01 s.e.m., t_22_ = -0.3, p = 0.76). We also tested reaction times since they often co-vary with confidence. Here, when the optimal confidence was lower, subjects took longer to respond to the prediction question (slope of reaction times vs. optimal confidence: -0.57±0.19 s.e.m., t_22_ = -3.07, p = 0.005), but not to the confidence question (slope: 0.04±0.08 s.e.m., t_22_ = 0.48, p = 0.64). However, this significant correlation is reducible to first-order estimates. We repeated the analysis previously reported for the subjects’ confidence but now with their reaction times: after regressing out several metrics derived from first-order estimates, the residuals of this regression no longer co-varied with optimal confidence (β = -0.103±0.124 s.e.m., t_22_ = -0.8, p = 0.41); and standardized regression coefficients indeed differed between the two regressions (paired difference of standardized βs: -0.233±0.080 s.e.m., t_22_ = -2.9, p = 0.008). In addition, there was no effect of streak type on (raw) reaction times both for the probability estimate and reports (paired difference between streak types, both p>0.27).

**Fig 5:**
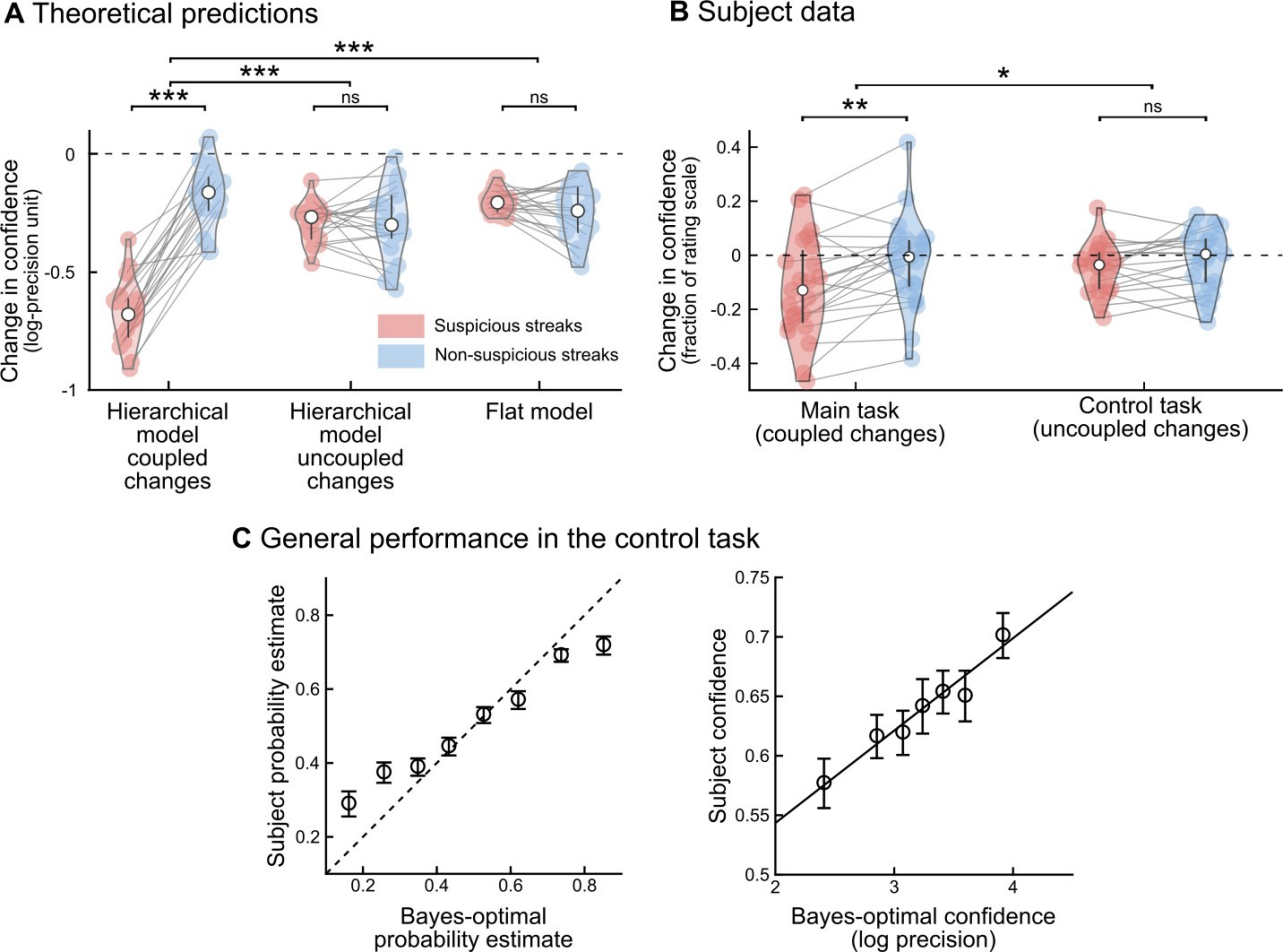
Control experiment: Subjects take into account the higher-order structure of the dynamics. In the control experiment, change points were uncoupled between the two transition probabilities, thereby abolishing the possibility to infer a change in one transition probability by only observing the other transition type. **(A)** Theoretical predictions for changes in confidence around the target streaks. The optimal hierarchical model for the main task assumes that change points are coupled (“hierarchical model, coupled changes”), which is no longer optimal in the case of uncoupled change points. This model was nevertheless used to identify suspicious and non-suspicious streaks and indeed it showed an effect of streak type on the change in confidence here in the control task as in the main task (Fig 4C). The optimal hierarchical Bayesian model for this control experiment is similar to this first model, the only difference is that it assumes that change points are uncoupled (“hierarchical model, uncoupled changes”). As expected, this model correctly showed no effect of streak type on the change in confidence. The flat model, by definition, ignores change points and therefore whether they are coupled or uncoupled, as a result it shows no effect of streak type (as in the main experiment). **(B)** Subjects showed no difference between streak types, like the hierarchical model for uncoupled changes. The results of the main task are reproduced from Fig 4C to facilitate visual comparison. **(C)** Subjects overall perform well in the control task, showing a tight agreement with the optimal hierarchical model for uncoupled change (the optimal model for this task) for both predictions (left) and confidence (right). In panels A and B, the error bars correspond to the inter-subject quartiles, distributions show subjects' data. In panel C, data points are mean ± s.e.m across subjects. In all panels; significance levels correspond to p = 0.048 (*), p<0.01 (**), p<0.001(***) in a two-tailed t-test.

A final alternative explanation for the effect shown in **Fig 4**is that suspicious streaks were more surprising and that subjects may become *generally uncertain* after surprising events. In this case, the effect would not reflect hierarchical inference but simply general surprise. We therefore performed a control experiment, in a different group of subjects, in which both probabilities changed independently (**Panel C in S2 Fig**): here, suspicious streaks were equally surprising but no longer signaled a *global* change point (**Fig 5A**). Indeed, generalization of a decrease in confidence was no longer observed for the hierarchical model with the correct task structure or in subjects (paired difference between suspicious and non-suspicious streaks: 0.03±0.02 s.e.m., t_20_ = 1.5, p = 0.15), see **Fig 5B**. This absence of an effect in the control task is significantly different from the effect found in the main task (difference of paired differences, two-sample t-test, t_42_ = -2.03, p = 0.048). This difference is not due poor performance in the control experiment (see **Fig 5C**): linear regression between the optimal hierarchical model for uncoupled change (the optimal model for this task) and subjects showed a tight agreement for both predictions (β = 0.61±0.06 s.e.m., t_20_ = 10.27, p = 2 10^−9^) and confidence (β = 0.08±0.01 s.e.m., t_20_ = 6.83, p = 1.2 10^−6^), as in the main task (see **Fig 3B and 3C**). The difference between the two tasks suggests an effect of higher-level factors (coupled vs. uncoupled change points) and thus further support the hierarchical model.

## Discussion

We have shown that previously proposed hallmarks of hierarchical processing are insufficient to categorically distinguish hierarchical from non-hierarchical models of learning in uncertain and changing environments, and we introduced a novel test to dissociate the two. The key features of our experiment are that subjects estimate multiple statistics that depend on the same change points, and that we analyze subjects’ confidence about their estimates. Our test taps into a unique property of hierarchical models: the ability to generalize between different probabilities that are coupled by the higher-order structure of the task. This ensured that hierarchical and flat accounts make *qualitatively different* predictions at individual trials. Based on both qualitative and quantitative model comparison, our results provide clear evidence for a hierarchical account. As such, they extend previous work that relied solely on quantitative model comparison or that did not test for hierarchy explicitly, and support the hierarchical learning framework that in recent years became highly influential to study cognition in both health and disease [2,4,10–12].

### Is confidence necessary to reveal the learner’s computations?

Choices and first-order reports are often used in behavioral science, but other metrics like a subject’s confidence and reaction times also proved useful to study cognition, see [31]. A feature that distinguishes our task from previous work on adaptive learning is the use of explicit confidence ratings to dissociate between flat and hierarchical models. Since the flat models considered here are known to provide very accurate first-order approximations to hierarchical (optimal) models [3,15,32], we reasoned that second-order estimates might prove useful as an additional source of information. Our simulations showed that even when first-order metrics are nearly indistinguishable, confidence was much less correlated between models. Although this conclusion holds both in a standard task in which one non-stationary statistic is learned (**Fig 2)** and in our more complex task (**S1 Fig**), we acknowledge that this does not guarantee that confidence is generally more diagnostic than first-order metrics. Rather, we take our experiment as an example case showing that second-order metrics can be worth studying in learning theory.

Importantly, while we believe confidence can be useful to discriminate hierarchical and flat models, we do not want to claim confidence is necessary (i.e. that it is impossible with other metrics). In particular, both the low correlations (**Fig 2)** and the critical dissociation between models (**Fig 4)** should in principle also be detectable using other, related metrics. Reaction times are one obvious candidate, as they are often an implicit measure of a subject’s confidence [33–36]. In our study, we found a correlation between reaction times and confidence, however, unlike for confidence reports, this correlation was reducible to first-order aspects such as the predictability of the next stimulus, and we found no effect of streak type that serves as our hallmark. Note that in our study, in contrast to accumulation [36] or waiting-time [35] paradigms, there is no general, principled reason for reaction times to co-vary with confidence. On the contrary, motor effects may even artifactually corrupt the relationship between confidence and reaction times since in our task reporting a more extreme estimate (typically associated with higher confidence) requires to move the response cursor further. This may explain why reaction times showed only a partial correlation with confidence and eventually did not show the hallmark of hierarchical inference.

Another interesting metric is the *apparent learning rate*. Previous studies reported modulations of the apparent learning rate by change points [2,5]. The optimal, hierarchical model indeed shows such modulations because its updates are confidence-weighted [4,24]: for a given prediction error, its updates are larger when confidence about prior estimates is lower, which is typically the case when a change point is suspected. However, we found that in simple experiments that require to monitor only the frequency of a stimulus or a reward, a flat model could exhibit similar modulations, which are therefore not diagnostic of hierarchical inference. In more complex experiments like the one here, the apparent learning rate could nevertheless show our signature of hierarchical inference. Theoretical analysis supports this hypothesis (see **Supplementary Results 5 in S1 File**) but we cannot assess it in our data, since this requires a trial-by-trial measure of the apparent learning rate, and thus trial-by-trial (not occasional) reports of first-order estimates. A trial-by-trial, model-free measure of the apparent learning rate is neither accessible if subjects make choices at each trial. In such studies [2,37], the authors could only use choices to compute an apparent learning rate in a sliding window of trials but this analysis lacks trial-by-trial resolution. In our task, investigating the apparent learning rate would require subjects to report their probability estimates after each trial, and hence to constantly interrupt the stimulus stream. This would probably interfere with the participants’ ability to integrate consecutive observations, which is critical for tracking transition probabilities, and therefore seems difficult to implement in practice. Furthermore, if an effect of streak type were observed on the apparent learning rate, it would probably be mediated by the subject’s confidence [4,24], in that case one may prefer to probe confidence directly.

We acknowledge that there are drawbacks of using confidence as the metric of interest. Although our simulations suggested that in theory confidence might discriminate flat and hierarchical models more reliably, in practice we found that the model fit between participants and the hierarchical, Bayes-optimal model was more precise for probability estimates than for confidence ratings (see **Figs 3B, 3C** and **5C**). This noisy character of confidence measurements was also reported previously [8,38–40] and may hinder the use of confidence as a metric to discriminate between models. This problem may be even worse when using an indirect indicator of confidence, such as reaction times.

We also acknowledge the possibility that asking subjects for explicit confidence reports about their inferences may have promoted the use of a sophisticated learning algorithm of a hierarchical nature. By contrast, simpler tasks in which subjects only make choices without being asked to reflect upon the reliability of their estimates, as we do here with confidence reports, could favor the use of simpler, possibly non-hierarchical learning algorithms. We cannot explore further this cautionary note in our current design because confidence reports are precisely key to detect the use of a hierarchical model here.

### Quantitative vs. qualitative model comparison and generality of the results

Quantitative model comparison is a widely used method for contrasting competing models. Following this approach, multiple models are fit onto the subjects’ data, and the model that achieves the best fit with respect to its complexity is deemed most likely [41,42]. This approach is attractive because it is generally applicable and it provides a common metric (e.g. goodness-of-fit, Bayes-Factor, exceedance probability) to compare different models. However, a limitation of quantitative model comparison is that it is not always clear what underlying factors are contributing to differences in fitness, and whether these factors are indeed most relevant to the question at hand. Moreover, quantitative model comparison only allows for relative conclusions, such as one model being *better than other tested models*, and is thus inherently restricted in scope to the models that are tested. Here, we used two models. The choice for the hierarchical model was straightforward: it is the optimal solution and therefore served as a benchmark. The choice for the flat model was motivated by both its resemblance to the classic, widely used delta rule with fixed learning rate [15,19], also see **Supplementary Results 1 in S1 File;** and because it is known to approximates the hierarchical model extremely well [3]. This renders it a worthy competitor, although not representative of all flat models.

A complementary approach is *qualitative* model comparison: analyzing specific, critical trials for the presence of *qualitative signatures* or *hallmarks* that uniquely identify or exclude one type of model. This approach is not only more transparent, but also enables more general conclusions, such as the *falsification* of one type of model [43]. Here, generality was granted by the fact that our test taps into a unique property of hierarchical models with an appropriate representation of the task structure. This argument derives from a priori principles, but we verified it by trying several parametrizations, priors and hypotheses of our models **(S2 Fig),** which supported that a model entertaining the correct dependencies can appropriately generalize. Importantly, we do not claim that the full Bayesian solution is the only model with this property. Other hierarchical models can be envisaged, such as two leaky accumulators (one per transition probability) coupled by a third mechanism that globally increases their leak whenever a change point is suspected in either accumulator.

Note that here, the quantitative and qualitative approaches were indeed complementary: quantitative model comparison provided evidence in favor of a hierarchical account, and the qualitative approach tested for unique hallmarks and thereby falsified a strictly flat account.

Last, we acknowledge that we falsify the flat account only in a strict sense: we rule out that humans *only* make use of a flat algorithm in our task. Our results cannot rule out the existence of flat learning algorithms in general. Several learning algorithms may co-exist and the brain may switch between strategies depending on context. Instead, the results show that humans are capable of hierarchical learning and that they use it even in a task that does not critically requires it, as shown by the near-optimal performance of the flat model. The human capacity of hierarchical learning is compatible with what others have suggested [4,6,10,21] and we provide a task for studying it.

### Implications for leaky integration and other implementations of adaptive learning

Our results falsify a strictly flat account of learning under uncertainty, therefore they also falsify the use of solely leaky integration by neural networks to solve adaptive learning tasks. Leaky integration is often deemed both biologically plausible and computationally efficient [3,18,19,37]. A sophisticated version of the leaky integration with metaplastic synapses allows partial modulation of the apparent learning of the network, without tracking change points or volatility [18]. Others have suggested that computational noise itself could enable a flat inference to automatically adapt to volatility [16,44]. Those approximate solutions dismiss the need to compute higher-level factors like volatility, they are thus appealing due to their simplicity; however, we believe that such solutions cannot explain the generalization afforded by hierarchical inference that we showed here. As such, at least for explicitly hierarchical tasks like the one studied here, such models have to be complemented to include higher-level factors.

One previously proposed bio-inspired model seems compatible with our result [6]. This model comprises two modules: one for learning and the other for detecting change points, or “unexpected surprise” [1]. When a change point is detected, a reset signal is sent to the learning module. Converging evidence indicates that noradrenaline could play such a role [45–48]. A global reset signal could promote learning for the two transition probabilities that are maintained in parallel in our task, thereby allowing the reset of both when only one arouses the suspicion of a change point. Such a hypothesis nevertheless needs to be refined in order to account for the fact that the two statistics can also be reset independently from one another, as in the control task.

### Learning and acting in a structured environment

Our task structure is more complex than many previous experiments which required to monitor only one generative statistic [2,5,7,10,27,49]. This may hamper translating our results to other experiments, but it has a certain ecological appeal since in real-life situations, multiple regularities are often embedded in a single context. We believe that more complex tasks are well-suited to distinguish complex computations and approximations thereof. Both are likely to be equivalent in simpler situations, whereas in highly structured environments with multiple interdependent levels [20,50], an effective learning algorithm can hardly obliviate the hierarchical nature of the problem to solve. Note that while we believe that hierarchically structured tasks are theoretically and practically *better suited* to test for hierarchical information processing, we do not claim that such tests are *impossible* in simpler tasks.

An interesting and difficult problem that we leave unaddressed here is how subjects may discover the task structure [20,51,52]. In our task, the optimal hierarchical model is able to correctly identify the current task structure (coupled vs. uncoupled change points), but only with moderate certainty even after observing the entire experiment presented to one subject (log-likelihood ratios range from 2 to 5 depending on subjects). Therefore, in principle, subjects who are not endowed with optimal computing power cannot identify reliably the correct structure from observations alone. We speculate that in real-life situations, some cues or priors inform subjects about the relevant dependencies in their environment; if true, then our experiment in which subjects were instructed about the correct task structure may have some ecological validity.

Interestingly, while the importance of hierarchical inference remains controversial in the learning literature [4,5,7,10,13,14,16–18,26,27], it seems more clearly established in the domain of decision making and action planning [50,53–57]. For instance, it was suggested that the functional organization of cognitive control is nested: low level cues trigger particular actions, depending on a stimulus-response association which is itself selected depending on a particular context [58]. In this view, negative outcomes may indicate that the (higher-level) context has changed and thus that a new rule now applies. This inference even seems to be confidence-weighted in humans: the suspicion of a change in context is all the stronger that subjects were confident that their action should have yielded a positive outcome under the previous context [59]. Those two studies feature an important aspect of hierarchy: a succession of (higher-level) task contexts separated by change points governs the (lower-level) stimuli. Our task also leverages another feature of hierarchy: it allows generalization and transfer of knowledge. A rule learned in a particular context can be applied in other contexts, for instance see [60,61]. Our results go beyond mere transfer: they show that the brain can *update* a statistic in the absence of direct evidence thanks to higher-level dependencies.

### Conclusion

In sum, we showed that previously proposed hallmarks are insufficient to distinguish hierarchical from non-hierarchical models of learning under uncertainty, and provided a new way to test between the two. Our results provide support to the hierarchical framework. Moreover, our work demonstrates the importance of using more complex task structures to test for hierarchy explicitly, and the usefulness of confidence as a source of information in learning theory. We believe our test can be applied beyond human learning to animal and computational models like neural networks, for which it may not be clear whether they make inferences that are hierarchical or not. As such, our test will be of interest to experimentalists and theoreticians alike.

## Materials and methods

### Participants

Participants were recruited by public advertisement. They gave a written informed consent prior to participating and received 20 euros for volunteering in the experiment. The study was approved by the local Ethics Committee (CPP n°08–021 Ile de France VII). 26 participants (17 females, mean age 23.8, s.e.m.: 0.49) performed the main task and 21 other participants performed the control task (11 females, mean age 23.0, s.e.m.: 0.59). We excluded participants who showed poor learning performance, which we quantified as the Pearson ρ coefficient between their probability estimates and the hierarchical, Bayes-optimal estimates. We used a threshold corresponding to 5% of the (lowest) values measured in this task (ρ<0.18, from a total of 105 participants in this study and others) This excluded 3 subjects from the main task, and none from the control task. Including those subjects does not change our main conclusion: regression of subject vs. optimal probability (resp. confidence): p = 3.3 10^–9^, (resp. p = 0.007); quantitative model comparison, with values fitted onto subject’s probability estimates (resp. confidence reports) supports the hierarchical model: p = 0.0003 (res. p = 0.050); effect of streak type of pre-post change in confidence: p = 0.047.

### Main task

The task was run using Octave (Version 3.4.2) and PsychToolBox (Version 3.0.11). Each participant completed a total of 5 blocks: 1 training block and 4 experimental blocks (2 auditory, 2 visual). Auditory and visual blocks alternated, with the modality of the first block randomised across participants. In each block, we presented binary sequences of 380 stimuli (1520 total) denoted A and B, which were either visual symbols or sounds and were perceived without ambiguity.

Sequences were generated according to the same principles as in previous studies [8,24]. A and B were randomly drawn based on two hidden transition probabilities which subjects had to learn. These probabilities were stable only for a limited time. The length of stable periods was randomly sampled from a geometric distribution with average length of 75 stimuli, truncated at 300 stimuli to avoid overly long stable periods. Critically, and contrary to other studies [2] the volatility was thus fixed (at 1/75). Transition probabilities were sampled independently and uniformly between 0.1–0.9, with the constraint that, for at least one of the two probabilities, the change in odds ratio (p/1-p) between consecutive stable periods was at least fourfold, thus guaranteeing that the change was effective. Across sequences and subjects, the actually used generative values indeed covered the transition probability matrix 0.1–0.9 uniformly, without any correlation (Pearson ρ = −0.0009, p = 0.98). Occasionally, the sequence was interrupted and subjects had to estimate the probability that the next stimulus would be either an A or a B and report their confidence in that estimate. Questions were located quasi-randomly, semi-periodically once each 15 stimuli on average (100 in total). Of the 100 questions, 68 questions were randomly placed; the remaining 32 questions were intentionally located just before and after 16 selected streaks (8 suspicious, 8 non-suspicious) and functioned as pre/post-questions to evaluate the effect of these streaks (see **Fig 4**). For details on the definition and selection of suspicious/non-suspicious streaks, see below.

To familiarize participants with the task they were carefully instructed and performed one training block of 380 stimuli (or ~12 minutes). To make sure they were fully aware of the volatile nature of the generative process, participants had to report when they detected changes in the hidden regularities. In the experimental blocks, reporting change points was omitted, but participants knew the underlying generative process was the same.

### Control task

The control task was very similar to the main one, with only two differences. (1) When a change occurred, it impacted only one of the two transition probabilities (randomly chosen). (2) During the training block, when subjects were required to report when they detected change points, they also reported which of the two transition probabilities had changed.

### Selection of sequences

Each randomly generated sequence was evaluated computationally and carefully selected to ensure that each subject encountered enough target moments during which the models make qualitatively different predictions, and that all sequences were balanced in terms of potential confounds such as streak duration and location. To this end, 4 random sequences of 380 stimuli long (each corresponding to one block) were analyzed computationally with the hierarchical and flat learning models, yielding 4 simulated ‘blocks’. The sequences, and associated trial-by-trial transition probability estimates from both models, were concatenated to form a single experimental sequence (of 1520 stimuli). This experimental sequence was then submitted to several selection criteria. First, we assessed whether the sequence contained at least 8 suspicious and 8 non-suspicious ‘streaks’. Consecutive repetitions were defined as ‘streaks’ if they consisted of at least 7 or more stimuli, and started after the 15th stimulus of a block. Streaks were classified as ‘suspicious’ if they aroused the suspicion of a change in the hierarchical, Bayes optimal model. Computationally, this was defined via the confidence in the probability of the observed repetition decreasing on average during the streak. To ensure the effect would be observable, only sequences in which the suspicious streaks led to a sizeable decrease in theoretical confidence levels were selected.

To control for factors that may potentially confound decreases in confidence, only sequences in which the average duration of suspicious and non-suspicious streaks was approximately identical, and in which there was at least one streak of each type in each block, were selected. In addition, subjects were not informed about the distinction between suspicious and non-suspicious streaks or that between random questions and pre-post questions that targeted the critical moments before and after streaks. Interviews performed after the experiment ruled out that subjects understood the goal of the experiment, as no subject had noticed that a sizable fraction (~30%) of questions purposefully targeted streaks.

### Learning models

The models used in this study are implemented in a Matlab toolbox available on GitHub and described in a previous publication [32]. The model termed “hierarchical” and “flat” here correspond respectively to the hidden Markov model (HMM) and the leaky integrator model in the toolbox. Here, we summarize the essential aspects of those models.

The hierarchical and flat models (M) are both ideal observer models, in the sense that they both use Bayes rule to infer what the posterior distribution of the statistic they estimate, *θ_t_*, should be, under a given set of assumptions. However, only for the hierarchical model, the set of assumptions actually corresponds to the generative process, rendering its estimates statistically optimal for the task. Nevertheless, both use Bayes rule to estimate the posterior distribution of *θ*_*t*_, based on a prior on this statistic and the likelihood provided by previous observations, *y*_*1*:*t*_ (here, a sequence of As and Bs):

$$p\left(\theta_{t}\text{|}y_{1:t},M\right)\propto p\left(y_{1:t}\text{|}\theta_{t},M\right)p\left(\theta_{t},M\right)$$ (Eq 1)

Subscripts denote the observation number within a sequence. In the main text, the models estimate the transition probabilities between successive stimuli, so that θ is a vector with two elements: θ = [p(A|A), p(B|B)]. Note that those two probabilities suffice to describe all transitions, since the others can be derived as p(B|A) = 1-p(A|A) and p(A|B) = 1-p(B|B). In **S3 Fig**, we also consider variants in which the model estimates another statistic, the frequency of stimuli: θ = p(A). Note that p(B) is simply 1-p(A).

The estimation of θ depends on the assumption of the ideal observer model (M). The flat model considers that θ is fixed, and evaluates its value based on a leaky count of observations. The internal representation of this model therefore has only one level: θ, the statistic of observations. When the true generative statistic is in fact changing over time, the leakiness of the model enables it to constantly adapt its estimate of the statistic and therefore to cope with changes. If the leakiness is tuned to the rate of change, the estimate can approach optimality (see **panel A in S1 Fig**).

By contrast, the hierarchical model entertains the assumption that θ can abruptly change at any moment. The internal representation of the model therefore has several levels beyond observations: a level characterizing the statistic of observations at a given moment (θ_t_) and a level describing the probability that of a change in θ occurs (p_c_). Conceivably, there could be higher-order levels describing changes in p_c_ itself [2]; however this sophistication is unnecessary here and we consider that p_c_ is fixed.

#### Flat model

The flat model assumes that the true value of θ is fixed, and it constantly infers its value given the evidence received. Therefore, the likelihood function can be decomposed as follows:

$$p\left(y_{1:t}\text{|}\theta \right)=p\left(y_{1}\text{|}\theta \right)\overset{t}{\underset{i=2}{\prod }}p\left(y_{i}\text{|}\theta ,y_{i-1}\right)=\frac{1}{2}\left[\theta_{A|A}^{N_{A|A}}{\left(1-\theta_{A|A}\right)}^{N_{B|A}}\right]\left[\theta_{B|B}^{N_{B|B}}{\left(1-\theta_{B|B}\right)}^{N_{A|B}}\right]$$ (Eq 2)

Where *N*_A|A_(t) denotes the number of AA pairs in the sequence *y*_1:t_. A convenient parametrization for the prior distribution is the beta distribution: p(θ) = Beta(θ_A|A_ | N^prior^_A|A_, N^prior^_B|B_). This parametrization allows for an intuitive interpretation of N^prior^_A|A_ and N^prior^_B|B_ as prior observation counts, and due to its conjugacy with the likelihood function (Eq2), inserting Eq2 into Eq1 yields that the posterior probability of θ is the product of two beta functions:

$$p\left(\theta \text{|}y_{1:t}\right)\propto \frac{1}{2}Beta\left(\theta_{A|A}\text{|}N_{A|A}+N_{A|A}^{prior},N_{B|A}+N_{B|A}^{prior}\right)Beta\left(\theta_{B|B}\text{|}N_{B|B}+N_{B|B}^{prior},N_{A|B}+N_{A|B}^{prior}\right)$$ (Eq 3)

We consider here that the count of observations (the number of AA, AB, BA and BB pairs) is leaky, so that observations that are further in the past have a lower weight than recent ones. We modeled this leakiness as an exponential decay ω, such that the k-th past stimulus has a weight e^-k/ω^:

$$N_{X|Y}\left(t\right)=\overset{t-1}{\underset{k=0}{\sum }}e^{-k/\omega }\delta_{y_{t-k},X}\delta_{y_{t-k-1},Y}$$ (Eq 4)

Where X and Y can be A or B, and δ_i,j_ is 1 if i = j and 0 otherwise. Note that a perfect integration, in which all observations are given the same weight, corresponds to the special case of ω being infinitely large. Also note that ω*ln(2) corresponds to the “half-life”, i.e. the number of observations after which the weight of a past observation is reduced by half.

In the main text (but **Fig 1**), we choose [N^prior^_A|A_, N^prior^_B|B_] = [0 0], for in this case, the mean estimate of the flat model becomes strictly equivalent to the estimate of a “delta rule” as the number of observations increases (see **Supplementary Result 1 in S1 File**). An alternative choice for the prior is the so-called Laplace-Bayes prior [1 1], which is uninformative in that it gives the same prior probability to any value of θ [41]. This choice is important for **Fig 1**, but not for the results in the main text (see **S3 Fig**).

#### Hierarchical model

The hierarchical model evaluates the current value of the generative statistic θ under the assumption that it may change at any new observation with a fixed probability p_c_. Note that, would the location of the change points be known, the inference of θ would be simple: one would simply need to count the number of pairs (*N*_A|A_, *N*_B|B_, *N*_A|B_, *N*_B|A_) since the last change point and apply Eq 2. However, without knowing the location of change points, one should in principle average the estimates given all possible locations of change points, which is in practice far too large a number. The computation is rendered tractable by the so-called Markov property of the generative process. If one knows θ at time *t*, then the next observation *y*_*t*+1_ is generated with θ_*t*+1_ = θ_*t*_ if no change occurred and with another value drawn from the prior distribution otherwise. Therefore, if one knows θ_*t*_, previous observations are not needed to estimate θ_*t*+1_. Casting the generative process as a Hidden Markov Model (HMM) enables to compute the joint distribution of θ and observations iteratively, starting from the prior, and updating this distribution by moving forward in the sequence of observations:

$$p\left(\theta_{t+1},y_{1:t+1}\right)=p\left(y_{t+1}\text{|}\theta_{t+1},y_{t}\right)\int p\left(\theta_{t},y_{1:t}\right)p\left(\theta_{t+1}\text{|}\theta_{t}\right)d\theta_{t}=p\left(y_{t+1}\text{|}\theta_{t+1},y_{t}\right)\left[\left(1-p_{c}\right)p\left(y_{1:t},\theta_{t}=\theta_{t+1}\right)+p_{c}\int p\left(y_{1:t},\theta_{t}\neq \theta_{t+1}\right)d\theta_{t}\right]$$ (Eq 5)

This integral can be computed numerically by discretization on a grid. The posterior probability can be obtained by normalizing this joint distribution.

**Probability reports and confidence ratings with the models.**

Both the flat and hierarchical models estimate a full posterior distribution for θ, therefore both models have a posterior uncertainty (or conversely, confidence) about their estimate. In that sense, the flat model can be considered as a delta rule that is extended to provide confidence estimates about first-order estimates (see **Supplementary Results 1 in S1 File** for more details about the flat model and delta rule).

The probability of the next stimulus (question #1 asked to subjects) was computed from the posterior using Bayes rule:

$$p\left(y_{t+1}\text{|}y_{1:t}\right)=\int p\left(y_{t+1}\text{|}\theta_{t+1},y_{t}\right)p\left(\theta_{t+1}\text{|}y_{1:t}\right)d\theta_{t+1}$$ (Eq 6)

Note that the first term in the integral, the likelihood, is nothing but the relevant transition probability itself (conditioned on the actual previous observation). This integral is therefore simply the mean of the posterior distribution of the relevant transition probability. The confidence in the reported probability estimate (question #2) was computed as the log-precision of this posterior distribution [8,24,29,62–64].

### Model fit

The flat and the hierarchical models have one free parameter each, respectively ω (the leakiness) and p_c_ (the prior probability of change point).

Unless stated otherwise, the analysis reported in the main text used the parameters that best fit the true probabilities used to generate the sequences of observations presented to subjects. More precisely, for each sequence of observations, we computed the probability of each new observation given the previous ones, as estimated by the models using Eq 5 and we compared it to the true generative probability. We adjusted the free parameters ω and p_c_ with grid-search to minimize the sum of squared errors (SSE) over all the sequences used for all subjects. The resulting values are ω = 20.3 and p_c_ = 0.014 (indeed close to the generative value 1/75).

We also fitted the parameters to the responses of each subject (**S3 Fig**). For probability estimates, the above grid-search procedure was repeated after replacing generative values with the subject’s estimates of probabilities at the moment of questions. For confidence reports, we used a similar procedure; note however that subjects used a bounded qualitative slider to report confidence whereas the model confidence is numeric and unbounded, so that there is not a direct mapping between the two. Therefore, the SSE was computed with the residuals of a linear regression between subject’s confidence and the model’s confidence. Here are the fitted values we obtained, reported as median (25–75 percentile): for the hierarchical model p_c_ = 0.0360 (0.0043–0.3728) when fitted onto probability estimates and p_c_ = 0.0134 (0.0006–0.0526) when fitted onto confidence reports: for the flat model, ω = 34.4 (25.9–100.5) and ω = 20.8 (8.2–50.2), respectively.

### Statistical analyses

All linear regressions between dependent variables (e.g. probability estimates, confidence ratings) and explanatory variables (optimal estimates of probabilities and confidence, surprise, prediction error, entropy) included a constant and were estimated at the subject level. The significance of regression coefficients was estimated at the group level with t-tests. For multiple regressions, explanatory variables were z-scored so that regression coefficients can be compared between the variables of a given regression. Unless stated otherwise, all t-tests are two-tailed.

## Supporting information

S1 Fig Correlation between the hierarchical and flat models is higher for probability estimates and than for confidence levels in our task. Panels **A** and **B** show the result of a simulation that is identical to **Fig 2**, except that here we simulated our task (see **Fig 3A**). The correlations indicate that probability estimates are nearly indistinguishable between the two models, whereas their confidence levels are more different. Note that the volatility level (0.013) and step size (4) used in the experiment are among the best values to discriminate between models on the basis of confidence. Those simulations used prior [1 1] for the flat model, but the results are qualitatively similar with prior [0 0] (see Methods). (**C**) The heatmap shows the histogram of confidence levels of the hierarchical and flat models in our task, i.e. for the sequences actually presented to subjects. Confidence levels appear relatively correlated (ρ = 0.58, consistent with panel **B**) but nevertheless dissociable. Our analysis targeted a particular class of diagnostic trials: red dots show the confidence levels on the trials corresponding the questions inserted after suspicious streaks, in which there is indeed no correlation between the two models (ρ = -0.12).
(TIF)

S2 Fig Comparison of different hierarchical task structures. In all panels, arrows indicate the conditional dependencies across hierarchical levels (plain lines) and time (dashed lines). The hierarchy comprises several levels: the binary observation y (level 1, bottom), the generative statistics of observations θ (level 2) and sudden changes C in those statistics (level 3) whose rate of occurrence depends on a volatility context (level 4, top). (A) Task by Behrens et al 2007: observations are governed by their frequency θ_A_. In this experiment, there are two volatility contexts (high, null) whereas volatility is fixed in our case. (B) Our main task, depicted in **Fig 3A**: observations are governed by transition probabilities between successive observations, θ_A|B_ and θ_B|A_ which share the same change points. (C) In our control task, observations are also governed by transition probabilities, but their change points are independent.
(TIF)

S3 Fig Theoretical predictions for various alternative models in the main task. Simulated changes in confidence around the target streaks in the main task. We consider three ideal observer models: the hierarchical model, the flat model with prior [0 0] and with prior [1 1]. Those models tracked either the transition probabilities between successive stimuli (**A**, **C**, **E**) or the frequency of stimuli (**B**, **D**, **F**). In the case of transition probabilities, we further distinguish between hierarchical models that assume that changes are coupled (i.e. identical, **S3 Fig**) or uncoupled (i.e. independent, **S2C Fig**) between the two transition probabilities. The free parameters of the models (the prior probability of change point p_c_ in the hierarchical model; the leak factor ω in the flat model) were fitted following three procedures: so as to provide the best estimation of the actual generative probabilities of the sequences at all trials (**A**, **B**) or the answers of subjects at the moment of all questions regarding probability estimates (**C**, **D**) or their confidence ratings (**E**, **F**). Panels **A** and **B** therefore show the results of models that were optimized to solve the probability estimation task. By contrast, panels **C**, **D**, **E**, **F**, show the results of models that were optimized to be as close as possible to subjects, which can in principle deviate from **A** and **B**.
Note that panel **A** corresponds to **Fig 4B**, expanded with two new models. None of the flat models in all plots shows an effect of streak type; some even predict increase (not decrease) in confidence (**B**, **D**, **F**). By contrast, a hierarchical model learning the stimulus frequency (**B**, **D**, **F**) seems more compatible with the subjects’ data: they indeed predict an effect of streak type. One could therefore wonder whether subjects actually monitor the item frequency, instead of transition probabilities in the task. Several pieces of evidence argue against this possibility (see **Supplementary Results 4**). In addition, this possibility is incompatible with the results of the control experiment (**Fig 5B**): a model that estimates a single statistic, namely the item frequency and the associated confidence, shows the same effect of streak type no matter whether change points are coupled between transition probabilities (main experiment) or uncoupled (control experiment). Last, note that a hierarchical model learning the transition probabilities shows an effect of streak type only if it assumes that change points are coupled (**A**, **C**, **D**).
Data points are mean ± s.e.m of model predictions for each subject; significance levels correspond to p<0.05 (*), p<0.01 (**), p<0.001(***) in paired t-test, except for comparisons involving the flat model with prior [0 0] in **F** for which we used a Wilcoxon sign rank test because of an outlier point (see large error bar).
(TIF)

S4 Fig Probability estimates show no effect of streak type. This figure is analogous to **Fig 4B and 4C**, except that it shows probability estimates reported at the moment of the pre/post streak questions, rather than the associated confidence levels. The error bars correspond to the inter-subject quartiles, distributions show subjects' data; the significance level ‘ns’ corresponds to paired t-tests with p>0.15.
(TIF)

S1 File Supplementary results. This file presents the result of supplementary analyses.
(PDF)

S2 File Full dataset. We provide the raw data as a Matlab data file. This data file can be read with Matlab, or a freely available software such as GNU Octave or Python. The file contains three cell variables: one for the subjects included in the main task, one for the subjects excluded from the main task, and one for the subjects (all included) in the control task. Each element of a cell corresponds to one subject, and the data are presented as a matrix. Each row is a trial, and the columns should be read as follows:
1. Sensory modality (“1”for visual, “0” for auditory)
2. Block number (1 to 4)
3. Observed binary sequence, coded as “1” and “2”
4. Generative probability of observing “1” when the previous stimulus is “2”
5. Generative probability of observing “2” when the previous stimulus is “1”
6. Subjects estimate of the probability of receiving “1” on the next trial, from 0 to 1 (with NaN when no question is asked)
7. Subject's confidence about the estimated probability, from 0 to 1 (with NaN when no question is asked)
8. Reaction times (s) for the probability report (with NaN when no question is asked).
Reaction time (s) for the confidence report (with NaN when no question is asked).
(MAT)

S3 File Code demo. This zipped folder contains Matlab code files showing how to compute the ideal observer models used in this article.
(ZIP)
