# Supplementary figures and images for: Confidence resets reveal hierarchical adaptive learning in humans
Source: PLoS Comput Biol. 2019 Apr 9;15(4):e1006972. doi: 10.1371/journal.pcbi.1006972 (PMC6474633; doi:10.1371/journal.pcbi.1006972)

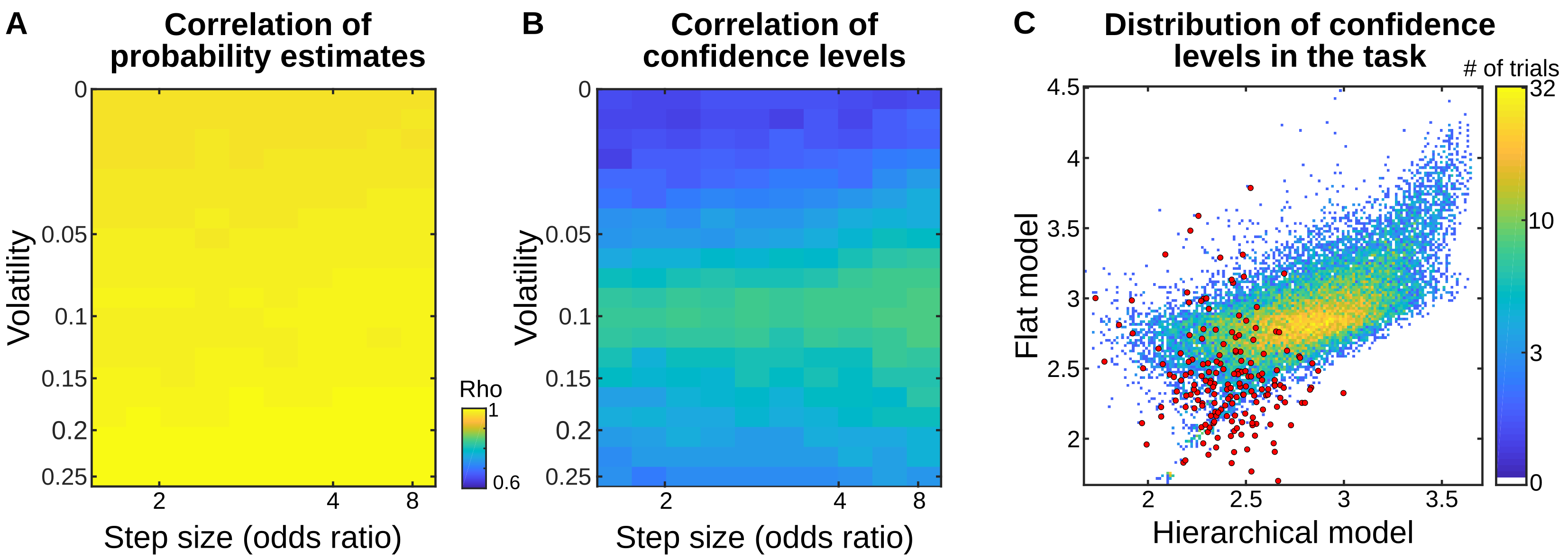

Supplement: S1 Fig — Panels A and B show the result of a simulation that is identical to Fig 2, except that here we simulated our task (see Fig 3A). The correlations indicate that probability estimates are nearly indistinguishable between the two models, whereas their confidence levels are more different. Note that the volatility level (0.013) and step size (4) used in the experiment are among the best values to discriminate between models on the basis of confidence. Those simulations used prior [1 1] for the flat model, but the results are qualitatively similar with prior [0 0] (see Methods). (C) The heatmap shows the histogram of confidence levels of the hierarchical and flat models in our task, i.e. for the sequences actually presented to subjects. Confidence levels appear relatively correlated (ρ = 0.58, consistent with panel B) but nevertheless dissociable. Our analysis targeted a particular class of diagnostic trials: red dots show the confidence levels on the trials corresponding the questions inserted after suspicious streaks, in which there is indeed no correlation between the two models (ρ = -0.12). (TIF) [file pcbi.1006972.s001.tif]

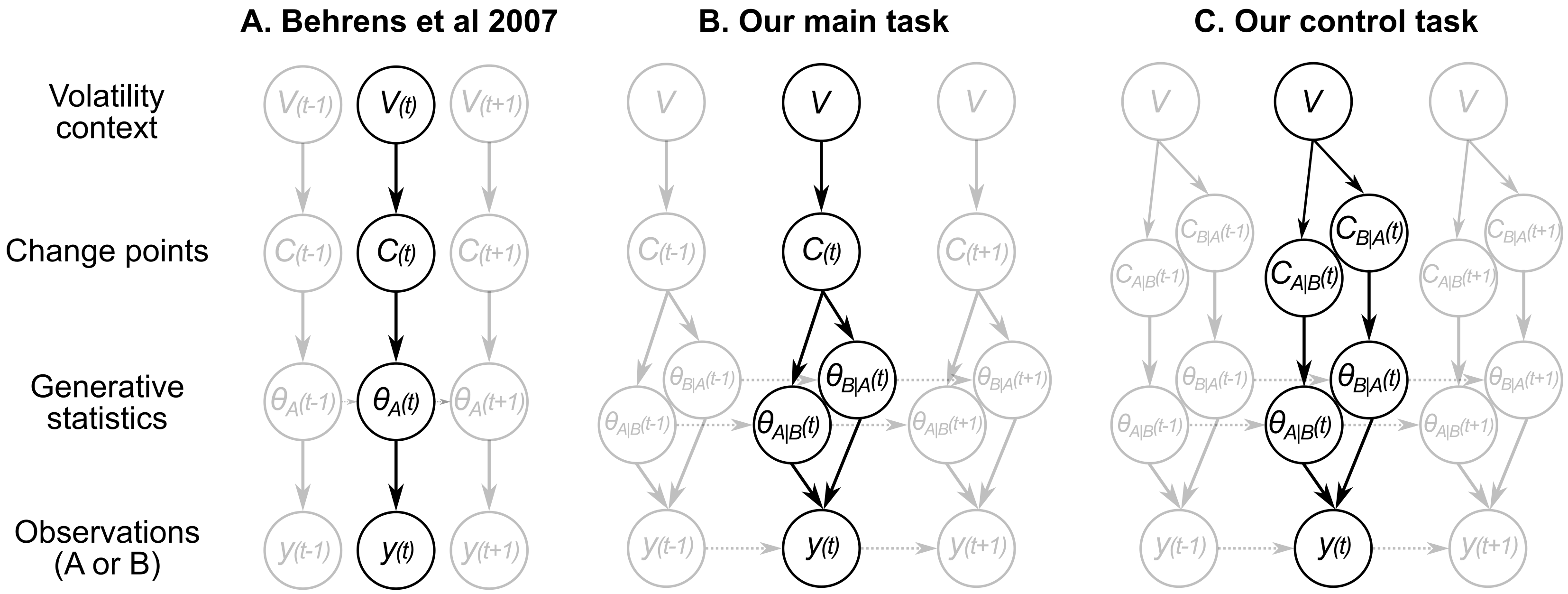

Supplement: S2 Fig — In all panels, arrows indicate the conditional dependencies across hierarchical levels (plain lines) and time (dashed lines). The hierarchy comprises several levels: the binary observation y (level 1, bottom), the generative statistics of observations θ (level 2) and sudden changes C in those statistics (level 3) whose rate of occurrence depends on a volatility context (level 4, top). (A) Task by Behrens et al 2007: observations are governed by their frequency θA. In this experiment, there are two volatility contexts (high, null) whereas volatility is fixed in our case. (B) Our main task, depicted in Fig 3A: observations are governed by transition probabilities between successive observations, θA|B and θB|A which share the same change points. (C) In our control task, observations are also governed by transition probabilities, but their change points are independent. (TIF) [file pcbi.1006972.s002.tif]

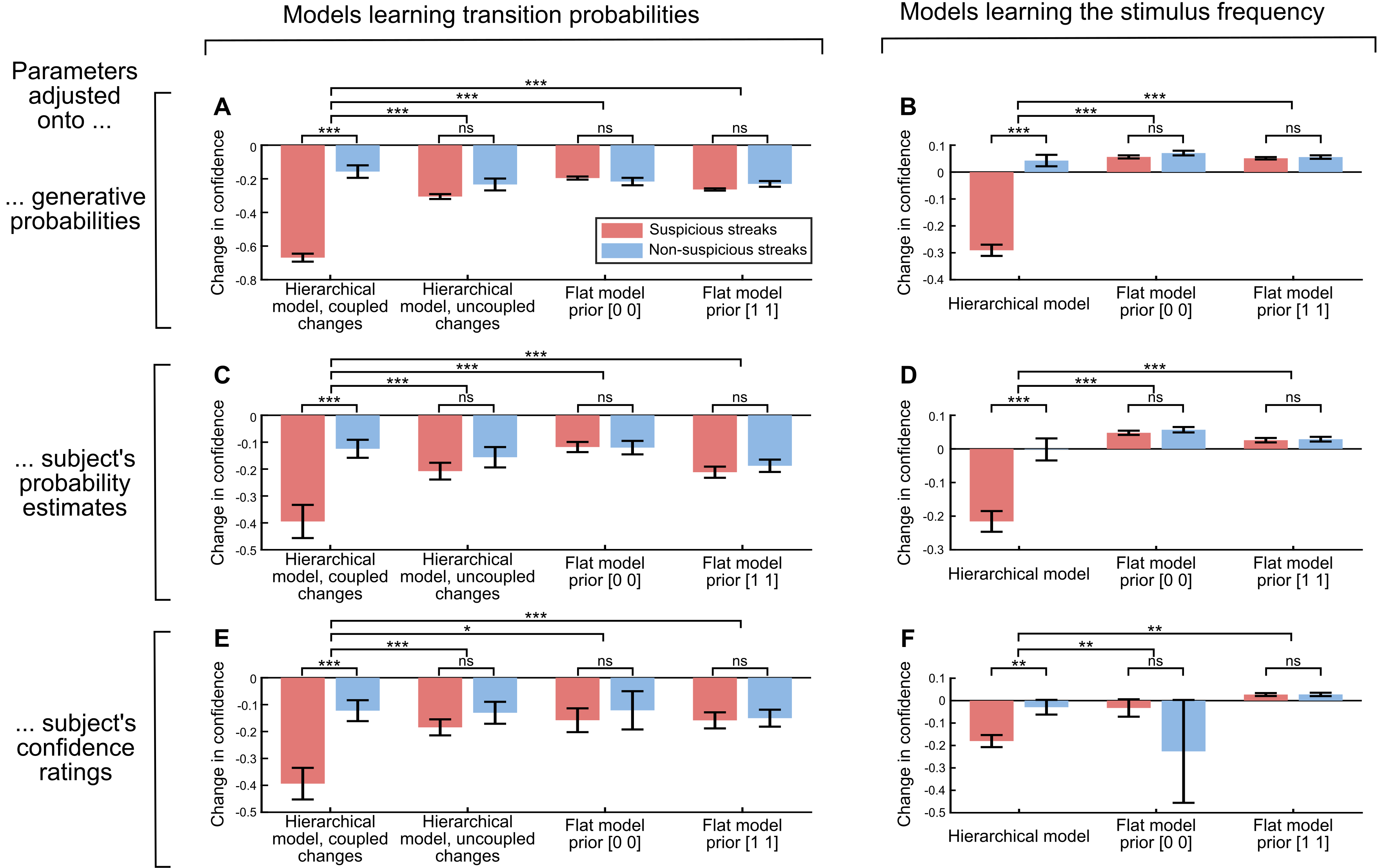

Supplement: S3 Fig — Simulated changes in confidence around the target streaks in the main task. We consider three ideal observer models: the hierarchical model, the flat model with prior [0 0] and with prior [1 1]. Those models tracked either the transition probabilities between successive stimuli (A, C, E) or the frequency of stimuli (B, D, F). In the case of transition probabilities, we further distinguish between hierarchical models that assume that changes are coupled (i.e. identical, S3 Fig) or uncoupled (i.e. independent, S2C Fig) between the two transition probabilities. The free parameters of the models (the prior probability of change point pc in the hierarchical model; the leak factor ω in the flat model) were fitted following three procedures: so as to provide the best estimation of the actual generative probabilities of the sequences at all trials (A, B) or the answers of subjects at the moment of all questions regarding probability estimates (C, D) or their confidence ratings (E, F). Panels A and B therefore show the results of models that were optimized to solve the probability estimation task. By contrast, panels C, D, E, F, show the results of models that were optimized to be as close as possible to subjects, which can in principle deviate from A and B. Note that panel A corresponds to Fig 4B, expanded with two new models. None of the flat models in all plots shows an effect of streak type; some even predict increase (not decrease) in confidence (B, D, F). By contrast, a hierarchical model learning the stimulus frequency (B, D, F) seems more compatible with the subjects’ data: they indeed predict an effect of streak type. One could therefore wonder whether subjects actually monitor the item frequency, instead of transition probabilities in the task. Several pieces of evidence argue against this possibility (see Supplementary Results 4). In addition, this possibility is incompatible with the results of the control experiment (Fig 5B): a model that estimates a single stat [file pcbi.1006972.s003.tif]

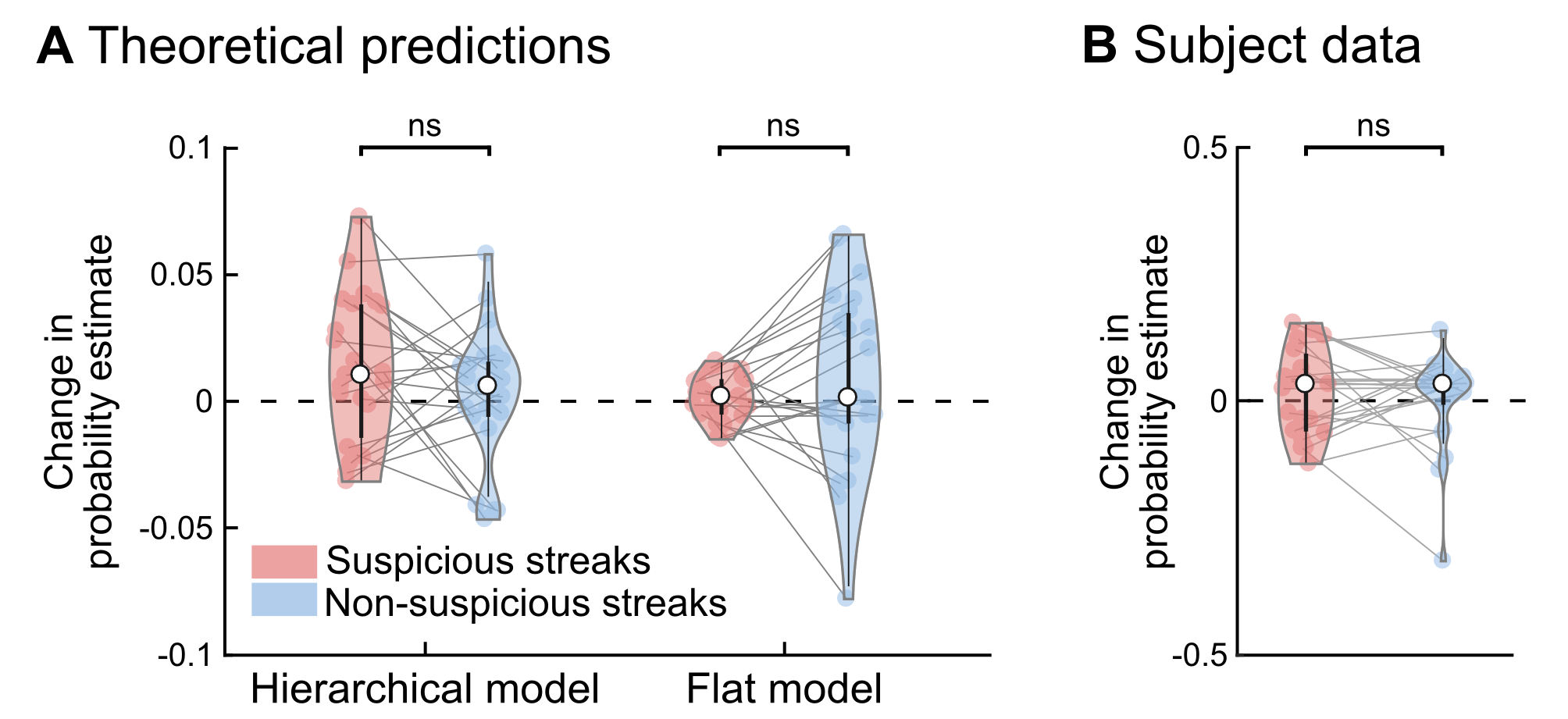

Supplement: S4 Fig — This figure is analogous to Fig 4B and 4C, except that it shows probability estimates reported at the moment of the pre/post streak questions, rather than the associated confidence levels. The error bars correspond to the inter-subject quartiles, distributions show subjects' data; the significance level ‘ns’ corresponds to paired t-tests with p>0.15. (TIF) [file pcbi.1006972.s004.tif]
