# Supplementary material for: Confidence resets reveal hierarchical adaptive learning in humans
Source: PLoS Comput Biol. 2019 Apr 9;15(4):e1006972. doi: 10.1371/journal.pcbi.1006972 (PMC6474633; doi:10.1371/journal.pcbi.1006972)
Supplement: S1 File — This file presents the result of supplementary analyses. (PDF) [file pcbi.1006972.s005.pdf]

Confidence resets reveal hierarchical adaptive learning in humans

Micha Heilbron and Florent Meyniel

SUPPLEMENTARY RESULTS

1 - Relation between the flat model and the delta rule

The equations of the flat model can be re-arranged so as to show the link with a leaky integrator, and hence, to a delta rule. For simplicity we derive those equations for the Bernoulli case (when one seeks to infer the frequency of items in a sequence), noting that the case of transition probabilities between successive items (p(A|A), p(B|B)) is nothing but the Bernoulli case when looking at each transition type separately (AA, BB).

Let's re-code the binary sequences as 1s and 0s, and estimate the probability $P_1(n)$ of observing a 1 after a sequence of observations $y_1, \dots, y_n$. $P_1(n)$ is the mean of a beta distribution, whose parameters are the (leaky) counts of observations and the prior count (cf. Eq. 3 and 5). Using the analytical solution for beta distributions, and recalling that the observation count is leaky, with exponential decay ω:

$$\begin{aligned} P_1(n) &= \frac{N_1 + N_1^{prior}}{N_1 + N_0 + N_1^{prior} + N_0^{prior}} \\ &= \frac{\sum_{t=1}^{n} y_t \left(e^{-1/\omega}\right)^{n-t} + N_1^{prior}}{\sum_{t=1}^{n} \left(e^{-1/\omega}\right)^{n-t} + N_1^{prior} + N_0^{prior}} \\ &= c \sum_{t=1}^{n} y_t \left(e^{-1/\omega}\right)^{n-t} + c\, N_1^{prior} \end{aligned} \qquad \text{(Eq 7)}$$

Where *c* can be approximated by a constant since $e^{-1/\omega}<1$ and *n* is typically large:

$$\begin{aligned} c &= \frac{1}{\frac{1-e^{-n/\omega}}{1-e^{-1/\omega}} + N_1^{prior} + N_0^{prior}} \\ &\approx \frac{1}{\frac{1}{1-e^{-1/\omega}} + N_1^{prior} + N_0^{prior}} \end{aligned}$$

A delta-rule with learning rate α reads as follow:

$$\begin{aligned} P_1(n) &= (1-\alpha)^n P_1(0) + \alpha \sum_{t=1}^{n} y_t (1-\alpha)^{n-t} \\ &\approx \alpha \sum_{t=1}^{n} y_t (1-\alpha)^{n-t} \end{aligned} \qquad \text{(Eq 8)}$$

Note that for large *n*, the term before the sum vanishes since $(1-\alpha)<1$, hence the approximation on the second line.

Comparison of Eq. 6 and 7 shows that the flat model and the delta rule are very similar, the only difference is that the leaky integration constantly adds a prior count in the flat model, whereas in the delta-rule, the impact of the starting point $P_1(0)$, which can be thought of as a prior, vanishes as more observations are accumulated. Stated differently, in the flat model, the inference progressively forgets about previous observations (like in the delta rule) and constantly factors in a prior about the estimated quantity (unlike the

delta rule). Note that with $[N^{prior}_1, N^{prior}_0] = [0\ 0]$, both models become asymptotically identical when *n* increases, making our 'flat' model equivalent to the delta rule.

2 - Apparent learning rate modulations in the flat model

Computing the apparent learning rate (the ratio between update $P_1(n)$-$P_1(n\text{-}1)$ and prediction error y_n-$P_1(n\text{-}1)$ leading to this update) shows, with a bit of math, that for typical choices of ω, $N^{prior}_1 > 0$ and $N^{prior}_0 > 0$, the apparent learning rate increases whenever two consecutive observations are identical, and decreases whenever they differ. Considering that consecutive observations are more likely to differ after a change point in the underlying generative probability, the learning rate of the flat model typically increases, on average, immediately after change points (see **Fig. 1**).

3 - Robustness of the results

Each subject performed four blocks, two with auditory stimuli and two with visual stimuli. We tested the robustness of the linear relations between subjects' and optimal values (**Fig. 3B-C**) by testing them separately in each block type. We found that the results were replicated in each sensory modality. For probability estimates, in the auditory modality β=0.69±0.07 s.e.m., t_{22}=9.27, p=4.7 10^{-9}; in the visual modality β=0.64±0.06 s.e.m., t_{22}=10.32, p=6.8 10^{-10}. For confidence, in the auditory modality β=0.10±0.04 s.e.m., t_{22}=2.85, p=0.009; in the visual modality β=0.09±0.03 sem, t_{22}=2.81, p=0.010. Interestingly, the regression coefficients were correlated across subjects between modalities (probability estimates: ρ_{23}=0.45, p=0.031; confidence ratings: ρ_{23}=0.81, p=2.6 10^{-6}), suggesting that inference in this task operates at an abstract, amodal level.

We further tested the robustness of the correlation between subjects' and optimal values by restricting the regression analysis to a subset of data points, namely, the questions that surround the target streaks (**Fig 4A**). The significant correlations were replicated on this subset of data for both probability estimates (β=0.57±0.07 s.e.m., t_{22}=7.6, p=1.4 10^{-7}) and confidence ratings (β=0.12±0.05 s.e.m., t_{22}=2.6, p=0.017).

We also tested the robustness of our central analysis of the effect of streak type on the change in confidence. In the main text, we report a dichotomy between suspicious and non-suspicious streaks, but in reality the 'suspiciousness' of a streak is a matter of degree: the more a streak arouses the suspicion of a change, the larger the decrease in confidence. We therefore regressed changes in subjective confidence onto the hierarchical model's changes in confidence across all streaks. In order to test whether the hierarchical model or the flat model provides a better account of the subjects' data, we also include the change in confidence of the flat model as a competing explanatory variable in the multiple regression. Regression coefficients were significant for the hierarchical model (β=0.07±0.02 s.e.m., t_{22}=3.7, p=0.001), but not for the flat model (β=0.01± 0.01 s.e.m., t_{22}=0.8, p=0.44), and the regression coefficients of the hierarchical model were significantly larger than those of the flat model (paired difference of βs=0.06±0.03 s.e.m., t_{22}=2.3, p=0.031), indicating that the hierarchical model provides a significantly better account of the subjects' data.

4 - Normative properties of confidence reports

In the ideal observer model, the answers to questions #1 and #2 in the task (probability estimate and confidence rating) are different readouts of the same posterior distribution, namely its mean and log-precision. If the subjects' answers to those questions also derive from the same inference process, then we expect that subjects who are closer to the optimal probability estimates are also those who are closer to the optimal confidence ratings. We therefore tested whether the linear regression coefficients (βs) linking subjects and the optimal hierarchical model were correlated between probability estimates and confidence ratings. The between-subject correlations was indeed significant: ρ_{23}=0.53, p=0.009.

We tested for further normative properties of the subjects' confidence ratings. Several factors, notably factors pertaining to first-order estimates, are expected to impact confidence ratings in this task from a normative viewpoint. We first show that those factors indeed impact the optimal confidence levels at the moments of questions during the task, and then report a similar analysis for the subjects' confidence ratings. Optimal confidence levels were entered into a multiple regression model which included the estimated probability itself, the entropy of this probability (which quantifies the estimated unpredictability of the next stimulus, it culminates when the estimated probability is 0.5), and the extent to which the current observation deviates from the previous estimate, as quantified by the surprise (negative log likelihood of the observations, (Shannon, 1948)) and the prediction error (one minus the likelihood of the current observation). Optimal confidence was lower when the estimated entropy was higher (β=-0.051±0.008, t_{22}=-6.1, p=4.2 10^{-6}), lower when surprise was larger (β=-0.142±0.026, t_{22}=-5.4, p=1.8 10^{-5}) and when prediction error was larger (β=-0.128±0.030, t_{22}=-4.2, p=3.5 10^{-4}).

To analyze subjects' confidence ratings, we added other explanatory variables to this multiple linear regression model, which correspond to subjects' estimates: the probability estimate, and the entropy corresponding to this estimate. Note that questions are asked only occasionally, so that we don't know the probability estimate of the subject at the *previous trial*, and therefore, we cannot compute the subjects' surprise and prediction error elicited by the last observation. Subjects' confidence was lower when the entropy of his estimate was higher (β=-0.125±0.009, t_{22}=-14.5, p=1.0 10^{-12}) and when the optimal surprise level was higher (β=-0.090±0.022, t_{22}=-4.1, p=5.0 10^{-4}).

Another aspect of subjects' accuracy is that their report of confidence is specific to the relevant statistics. In the experiment, subjects monitor two transition probabilities, there are therefore two confidence levels, each being attached to one transition type. Questions asked subjects to estimate the likelihood of the next stimulus, which depends on only one of the two transition probabilities: the one that is relevant given the identity of the previous stimulus at the moment of the question. We estimated a multiple linear regression in which the subjects' confidence ratings were regressed onto both the optimal relevant confidence levels, and the optimal irrelevant confidence level (those attached to the irrelevant transition). The regression coefficients corresponding to the relevant confidence levels were significant (β=0.039±0.014 s.e.m., t_{22}= 2.8, p=0.011), those for the irrelevant confidence were not (β=0.001±0.008 s.e.m., t_{22}=0.1, p=0.89) and the difference between the two was significant (paired difference of βs=0.038±0.018 s.e.m., t_{22}=2.0, p=0.027,

one-tailed test). A different multiple linear regression indicates, in addition, that confidence ratings are selectively modulated by the (optimal) entropy of the relevant transition probability, as opposed to the irrelevant one (paired difference of βs=-0.020±0.007 s.e.m., t_{22}=-2.9, p=0.008). Optimal confidence levels show the same effect: replacing the subjects' confidence ratings in this latter regression with the optimal confidence levels also reveals a significant difference (paired difference of βs=-0.050±0.016, t_{22}=-3.2, p=0.0045). By contrast, such a difference is not observed when replacing the subjects' confidence ratings with the optimal confidence of a model that monitors solely the frequency of items (paired difference of βs=0.005±0.008, t_{22}=0.6, p=0.54). Together, those results indicate that subjects reported specifically the confidence attached to the transition probability relevant at the moment of the question.

5 - Theoretical effects on the apparent learning rate

We cannot assess the apparent learning rate of subjects on a trial-by-trial basis here since it would require that subjects report their first-order estimates on every trial, whereas they did it only occasionally. However, we can run such an analysis on our simulated models. We found a specific effect of streak type on the apparent learning rate of the hierarchical model, which increased more after suspicious streaks than non-suspicious ones (0.11±0.01 s.e.m., p=2.9 10^{-14}, t_{22}=17.2), there was no difference in the flat model (-0.0023±0.0022 s.e.m., p=0.3, t_{22}=-1.1) and the difference between models was significant (paired difference of differences, -0.11±0.01 s.e.m., p=1.4 10^{-14}, t_{22}=-17.9). This effect of streak type in the hierarchical model for uncoupled change points was no longer observed in the control task with uncoupled change points (-0.001±0.005 s.e.m., p=0.85, t_{20}=-0.2). In other words, the apparent learning rate passes the test we propose to detect the use of a hierarchical model.
